# Moderate ethanol exposure disrupts energy homeostasis between central and peripheral system in APP/PS1 mice

**DOI:** 10.1186/s13041-025-01192-z

**Published:** 2025-03-17

**Authors:** Shinwoo Kang, Jeyeon Lee, Paul H. Min, Doo-Sup Choi

**Affiliations:** 1 Department of Molecular Pharmacology and Experimental Therapeutics, Rochester, MN 55905 USA; 2 Department of Pharmacology College of Medicine, Soonchunhyang University, Cheonan-si, 31151 Republic of Korea; 3 Department of Radiology, Mayo Clinic College of Medicine and Science, Rochester, MN 55905 USA; 4 Department of Biomedical Engineering, College of Medicine, Hanyang University, Seoul, Republic of Korea; 5 Department of Psychiatry and Psychology, Mayo Clinic College of Medicine and Science, Rochester, MN 55905 USA

## Abstract

To investigate the effects of moderate ethanol exposure on glucose metabolism in APP/PS1 mice, an early-onset Alzheimer’s disease (AD) mouse model, we employed an fluoro-deoxy-glucose (FDG)-micro-positron emission tomography (PET). We also utilized the comprehensive lab animal monitoring system (CLAMS) to measure whole-body energy expenditure and respiratory exchange ratio (RER). We found that ethanol exposure increased glucose metabolism in the brain as measured by FDG-PET. Also, CLAMS data indicated a decrease in RER, suggesting a shift toward fat utilization as the primary energy source. Following ethanol exposure in APP/PS1 mice, these findings reveal a distinct metabolic difference between brain and peripheral tissues.

**Supplementary Information:**

The online version contains supplementary material available at 10.1186/s13041-025-01192-z.

## Main text

AD is characterized by metabolic dysfunction both centrally, in the brain, and peripherally, affecting overall energy homeostasis. One hallmark of AD is impaired glucose metabolism, which can be observed using FDG-PET imaging, particularly in regions like the cortex and hippocampus [1, 2]. Moderate ethanol exposure is known to influence both brain activity and systemic metabolism, yet the effects of ethanol exposure on metabolism in an AD context still need to be explored [3–5].

We examined ethanol’s effects on brain and peripheral metabolism in APP/PS1 mice, an AD mouse model [4]. By combining FDG-PET imaging to assess brain glucose metabolism with CLAMS to monitor whole-body energy utilization, it seeks to elucidate the differential effects of ethanol on brain and peripheral metabolism and investigate whether ethanol-induced changes in brain glucose metabolism correlate with shifts in systemic energy balance.

**Fig. 1:**
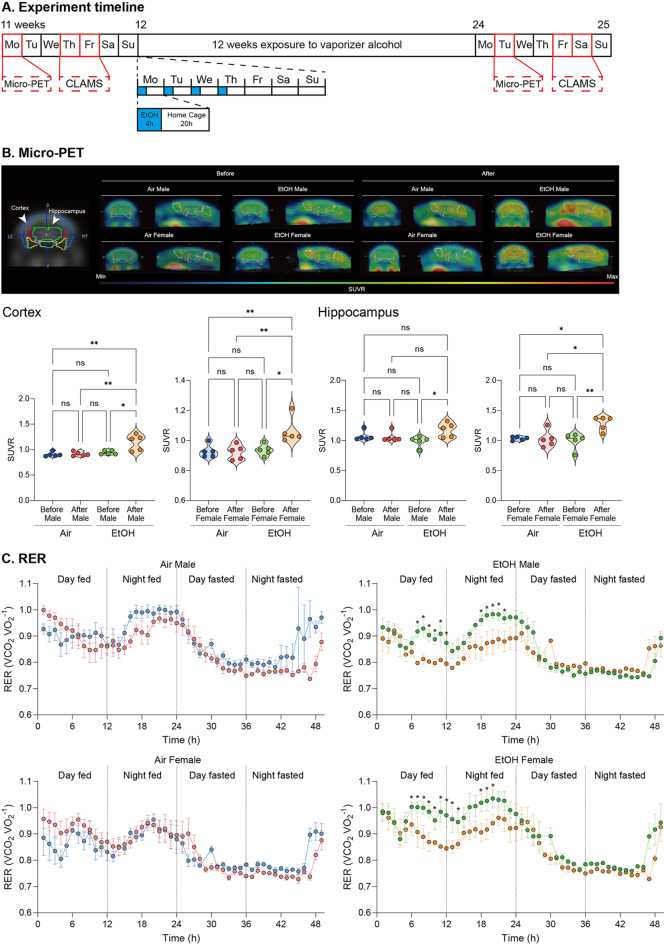
Effects of chronic ethanol exposure on brain and peripheral metabolism in AD mice. (**A**) Mice were exposed to ethanol vapor for 12 weeks, and baseline and post-exposure metabolic assessments were performed using FDG-PET for brain glucose metabolism and CLAMS for peripheral energy expenditure. (**B**) Representative FDG-PET images showing increased glucose uptake in the cortex and hippocampus following ethanol exposure. (**C**) Ethanol exposure also significantly decreased the respiratory exchange ratio (RER). Data represent mean ± SEM; *n* = 5 per group. *P < 0.05 comparing each group. See Table S1 for full statistical information

To examine the relevance of ethanol exposure-induced brain glucose metabolism changes to whole-body energy utilization changes in an AD mouse model (pre- vs. post-ethanol exposure), mice were exposed to ethanol at 12–24 weeks of age. FDG-PET and CLAMS were measured before ethanol exposure and at 11 ~ 12 and 24 ~ 25 weeks of age. Male and female APP/PS1 mice were exposed to ethanol vapor or room air using a vapor chamber for 4 h from 9:00 AM to 1:00 PM, followed by exposure to room air for 20 h. Ethanol vapor exposure was conducted under standardized laboratory conditions, and blood ethanol concentration (BEC) was measured at an average of 170 mg/dl (Fig. S1A). This process was repeated 4 consecutive days per week, with 3 days (withdrawal period) spent in the home cage to investigate the long-term effects of ethanol exposure (Fig. 1A) [4]. Additionally, no significant differences in body weight were observed between the Air and EtOH groups during the 12-week exposure period (Fig. S1B).

FDG-PET scans revealed significant changes in brain glucose metabolism following ethanol exposure. Figure 1B shows representative standard uptake value ratio (SUVR) images of the cortex and hippocampus before and after ethanol exposure. The SUVR images indicates increased glucose uptake in the hippocampus and cortex, key AD pathology regions. In the cortex, ethanol exposure led to a substantial increase in glucose uptake. Mice showed significantly higher SUVR values after ethanol exposure than pre-exposure (*p* < 0.01), indicating enhanced metabolic activity in this region. The cortex, a key area affected by AD, displayed a marked response to ethanol, suggesting that ethanol may stimulate brain activity in early-stage AD. Similarly, in the hippocampus, glucose metabolism increased significantly after ethanol exposure (*p* < 0.05). The hippocampus, heavily implicated in memory and cognitive function, showed enhanced glucose uptake post-exposure, indicating a potential acute metabolic boost from ethanol exposure (Table S1). Both male and female APP/PS1 mice were included in this study, and initial statistical analyses indicated no significant interaction between sex and treatment (Fig. S1C).

CLAMS data in Fig. 1C show significant changes in peripheral metabolism before and after ethanol exposure. The RER, which reflects the balance between carbohydrate and fat metabolism, decreased notably following ethanol exposure. Before ethanol exposure, RER values indicated a relatively balanced use of carbohydrates and fats for energy, with higher RER values during feeding periods. After ethanol exposure, a significant reduction in RER was observed, particularly during the day and night phases. This decrease suggests a shift toward greater fat oxidation as the primary energy source after moderate ethanol exposure (*p* < 0.05). These metabolic shifts, characterized by a decrease in RER, suggest that ethanol exposure triggers a reliance on fat metabolism in peripheral tissues, likely due to the increased energy demands of ethanol metabolism and its effects on liver function. The reduction in RER values was consistent across feeding and fasting periods, indicating a persistent shift in peripheral energy balance (Table S1).

The contrasting results from FDG-PET and CLAMS highlight the complex metabolic interplay between the brain and peripheral tissues following ethanol exposure in an AD model. The increased brain glucose metabolism observed via PET may be attributed to ethanol stimulatory effects on neurotransmission and neuronal glucose uptake [6, 7]. However, the simultaneous decrease in RER suggests that peripheral tissues, particularly the liver and muscle, may compensate by shifting to fat oxidation, likely due to the energy demands of ethanol metabolism and its effect on hepatic lipid oxidation [8].

This metabolic disconnection, where the brain exhibits increased glucose uptake while peripheral tissues favor fat utilization, raises important questions about the systemic effects of ethanol in AD. This dissociation suggests ethanol disrupts central and peripheral energy homeostasis. The enhanced brain glucose uptake may offer short-term benefits, such as increased neuronal activity. However, the concurrent shift in peripheral metabolism could indicate systemic stress, particularly in organs like the liver, which are responsible for ethanol metabolism [4]. One potential explanation is that ethanol alters central and peripheral energy homeostasis through distinct mechanisms, with the brain prioritizing glucose for rapid energy needs. In contrast, peripheral tissues adjust to the energetic cost of metabolizing ethanol [9, 10]. The limitation of our studies includes the lacking wild-type control and potential confounding caloric effects of ethanol in CLAMS experiment.

Moderate ethanol exposure in an AD mouse model increases brain glucose metabolism and a concurrent shift toward peripheral fat oxidation. These results suggest a metabolic dissociation between brain and peripheral energy regulation, providing new insights into the complex role of metabolic dysfunction in AD. Ethanol-induced metabolic dissociation may involve AMPK and insulin signaling pathways. AMPK activation in peripheral tissues promotes fat oxidation, aligning with the observed reduction in RER, while its activation in the brain enhances glucose uptake, explaining the increased SUVR in FDG-PET scans [11].

Given that AUD has been linked to cognitive decline and increased amyloid accumulation in aging populations, particularly those over 65 [12], future research should investigate how these changes exacerbate disease progression and whether similar metabolic shifts occur in human AD patients.

## Electronic supplementary material

Below is the link to the electronic supplementary material.

Supplementary Material 1

## Data Availability

All data generated and/or analyzed during this study are included in this published article and its supplementary information. (Zenodo: https://zenodo.org/records/14969159).

## Abbreviations

AD: Alzheimer’s disease
FDG: Fluorodeoxyglucose
PET: Positron emission tomography
CLAMS: Comprehensive Lab Animal Monitoring System
RER: RRespiratory exchange ratio
SUVR: Standard uptake value ratio
