# Supplementary material for: Moderate ethanol exposure disrupts energy homeostasis between central and peripheral system in APP/PS1 mice
Source: Mol Brain. 2025 Mar 17;18:21. doi: 10.1186/s13041-025-01192-z (PMC11912666; doi:10.1186/s13041-025-01192-z)
Supplement: Supplementary file 1 — Supplementary Material 1 [file 13041_2025_1192_MOESM1_ESM.docx]

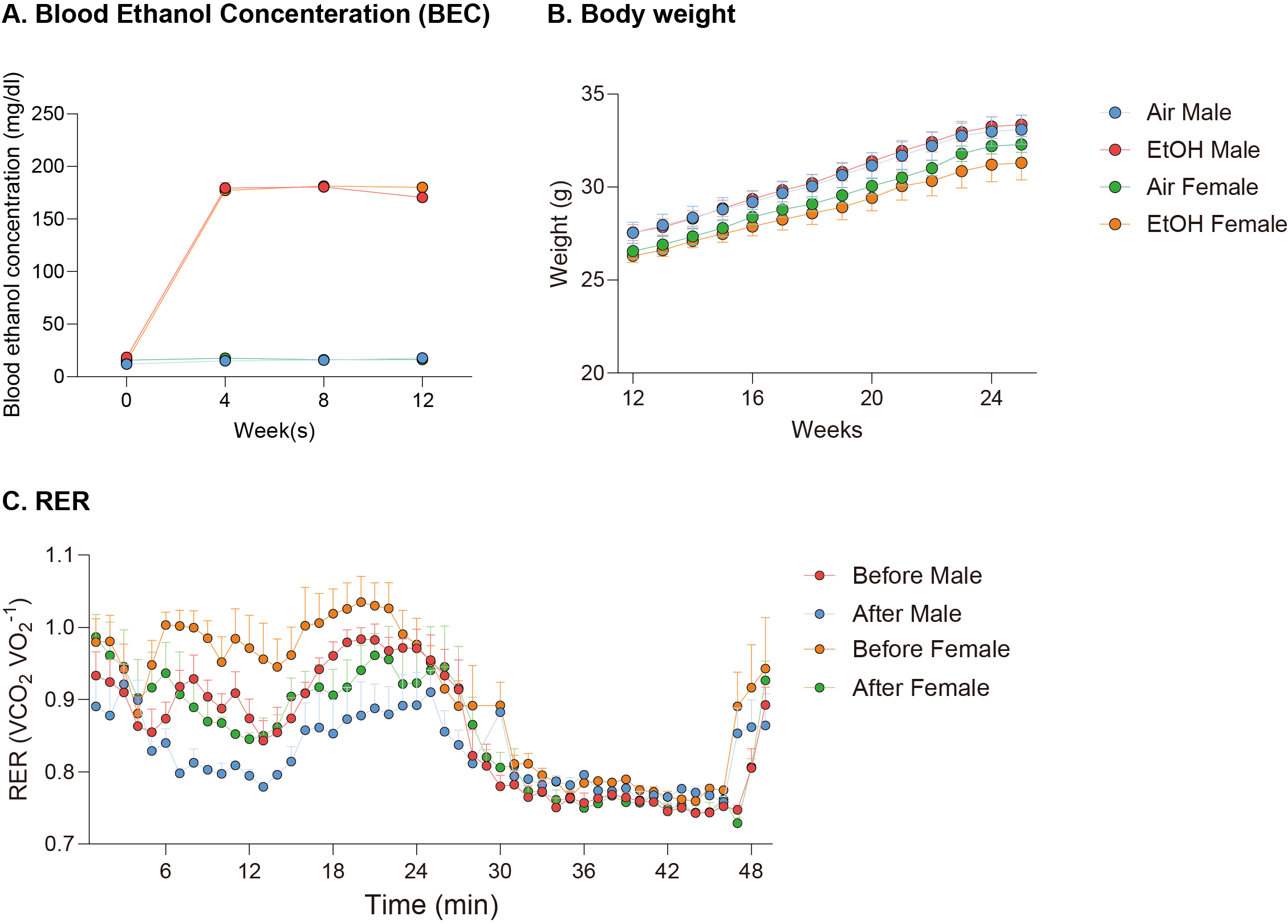


**Supplementary Figure 1. Measurements of body weight, blood ethanol levels, and RER in APP/PS1 mice during ethanol exposure.** (A) In APP/PS1 mice, body weight and blood ethanol concentration (BEC) were measured every four weeks throughout the ethanol exposure period. (B) In APP/PS1 mice, both body weight during ethanol exposure and blood alcohol concentration (BAC) were recorded every four weeks. (C) Ethanol exposure significantly decreased RER in both male and female mice. However, there was no significant difference between males and females. Data are presented as mean ± SEM with a sample size of 5–10 per group. For complete statistical details, please refer to Table 1.

**Table 1. Summary of statistical analysis**

| **Figure** | | **Statistical Tests** | **Comparison** | **Value** | **P value** |
| --- | --- | --- | --- | --- | --- |
| **Figure.1** | **B**  **(Cortex)** | Tukey's multiple comparisons test | Before  Male(Air) vs. After  Male(Air) | q=0.190 | P=0.9992 |
|  |  | Tukey's multiple comparisons test | Before  Male(Air) vs. Before Male(EtOH) | q=0.9665 | P=0.9019 |
|  |  | Tukey's multiple comparisons test | Before  Male(Air) vs. After  Male(EtOH) | q=6.152 | P=0.0025 |
|  |  | Tukey's multiple comparisons test | After  Male(Air) vs. Before Male(EtOH) | q=0.7875 | P=0.9433 |
|  |  | Tukey's multiple comparisons test | After  Male(Air) vs. After  Male(EtOH) | q=5.973 | P=0.0032 |
|  |  | Tukey's multiple comparisons test | Before Male(EtOH) vs. After  Male(EtOH) | q=5.186 | P=0.0101 |
|  |  | Tukey's multiple comparisons test | Before  Female(Air) vs. After  Female(Air) | q=0.2182 | P=0.9986 |
|  |  | Tukey's multiple comparisons test | Before  Female(Air) vs. Before Female(EtOH) | q=0.3428 | P= 0.9948 |
|  |  | Tukey's multiple comparisons test | Before  Female(Air) vs. After  Female(EtOH) | q=5.345 | P= 0.0080 |
|  |  | Tukey's multiple comparisons test | After  Female(Air) vs. Before Female(EtOH) | q=0.5610 | P= 0.9781 |
|  |  | Tukey's multiple comparisons test | After  Female(Air) vs. After  Female(EtOH) | q=5.564 | P= 0.0059 |
|  |  | Tukey's multiple comparisons test | Before Female(EtOH) vs. After  Female(EtOH) | q=5.003 | P= 0.0131 |
|  | **B**  **(Hippocampus)** | Tukey's multiple comparisons test | Before  Male(Air) vs. After  Male(Air) | q=0.3080 | P=0.9962 |
|  |  | Tukey's multiple comparisons test | Before  Male(Air) vs. Before Male(EtOH) | q=2.005 | P=0.5068 |
|  |  | Tukey's multiple comparisons test | Before  Male(Air) vs. After  Male(EtOH) | q=2.497 | P=0.3248 |
|  |  | Tukey's multiple comparisons test | After  Male(Air) vs. Before Male(EtOH) | q=1.697 | P=0.6354 |
|  |  | Tukey's multiple comparisons test | After  Male(Air) vs. After  Male(EtOH) | q=2.805 | P=0.2349 |
|  |  | Tukey's multiple comparisons test | Before Male(EtOH) vs. After  Male(EtOH) | q=4.502 | P0.0267 |
|  |  | Tukey's multiple comparisons test | Before  Female(Air) vs. After  Female(Air) | q=0.1477 | P0.9996 |
|  |  | Tukey's multiple comparisons test | Before  Female(Air) vs. Before Female(EtOH) | q=0.7352 | P=0.9531 |
|  |  | Tukey's multiple comparisons test | Before  Female(Air) vs. After  Female(EtOH) | q=4.902 | P=0.0151 |
|  |  | Tukey's multiple comparisons test | After  Female(Air) vs. Before Female(EtOH) | q=0.5875 | P=0.9750 |
|  |  | Tukey's multiple comparisons test | After  Female(Air) vs. After  Female(EtOH) | q=5.049 | P=0.0123 |
|  |  | Tukey's multiple comparisons test | Before Female(EtOH) vs. After  Female(EtOH) | q=5.637 | P=0.0053 |
|  | **C**  **(Air Male)** | Two-way ANOVA | Air Male(Before) vs. Air Male(After) | F(48,338) = 7.137 | P<0.0001 |
|  | **C**  **(EtOH Female)** | Two-way ANOVA | EtOH Male(Before) vs. EtOH Male(After) | F(48, 290) = 10.82 | P<0.0001 |
|  | **C**  **(Air Male)** | Two-way ANOVA | Air Female(Before) vs. Air Female(After) | F(48,338) = 9.264 | P<0.0001 |
|  | **C**  **(EtOH Female)** | Two-way ANOVA | EtOH Female(Before) vs. EtOH Female(After) | F(48,338) = 13.57 | P<0.0001 |
| **Supplementary**  **Figure. 1** | **A** | Two-way ANOVA | Air vs. EtOH | F(3, 128) = 1629 | P<0.0001 |
|  | **C** | Two-way ANOVA | Before Male vs. Before Female | q=7.113 | P<0.0001 |
|  |  |  | After Male vs. After Female | q=1.430 | P=0.7430 |
